# A modified tunnel technique to treat multiple gingival recessions: Case Series

**DOI:** 10.1590/0103-6440202305502

**Published:** 2023-07-17

**Authors:** Dennis Malta Guimarães, Patrícia Freitas de Andrade, Julio Rebollal, André Martins das Neves, José Mauro Granjeiro

**Affiliations:** 1 Latin American Institute of Dental Research and Education (ILAPEO), Department of Implantology, Curitiba, Paraná, Brazil; 2 School of Dentistry of Ribeirão Preto, University of São Paulo, Ribeirão Preto, São Paulo, Brazil; 3 School of Dentistry, Federal University of Minas Gerais, Belo Horizonte, Minas Gerias, Brazil

**Keywords:** root coverage, connective tissue graft, gingival recession, tunnel technique, periodontal disease

## Abstract

This case series reports a modified tunnel technique with connective tissue graft for the root coverage of multiple Miller Class I, II, and III gingival recessions. The modified approach presents an innovative suture technique to improve the stability and position of the graft. Ten patients with multiple gingival recessions (n=85 teeth) received surgical root coverage treatment. The gingival recession height and width were measured and presented as median, minimum, and maximum values. The percentage of the root coverage after at least 12 months expressed the treatment effectiveness. The Shapiro-Wilk test evaluated the normality; pared Wilcoxon test determined the exact P-value for the differences in the height of the gingival recession before and after surgical treatment (α = 0.05). An average of 97.9% (± 5.6%, p < 0.0001) root coverage after treatment occurred, and 73 out of 85 recessions presented complete root coverage after 12 months. Treatment of Miller class I and II gingival recessions resulted in root coverage higher than 99 and class III higher than 95% (p < 0.0001). The presented case series report the efficacy of a modified surgical technique promoting more than 95% of root coverage after 12 months in multiple Miller Class I, II, and III gingival recessions. Well-designed blind randomized controlled trials are needed to validate the proposed technique.

## Resumo

Esta série de casos relata uma técnica de túnel modificada com enxerto de tecido conjuntivo para o recobrimento radicular de múltiplas recessões gengivais Classe I, II e III de Miller. A abordagem modificada apresenta uma técnica de sutura inovadora para melhorar a estabilidade e a posição do enxerto. Dez pacientes com múltiplas recessões gengivais (n=85 dentes) receberam tratamento cirúrgico de recobrimento radicular. A altura e a largura da recessão gengival foram mensuradas e apresentadas como valores medianos, mínimos e máximos. A eficácia do tratamento foi expressa como uma porcentagem da cobertura radicular após pelo menos 12 meses. O teste de Shapiro-Wilk avaliou a normalidade; o teste de Wilcoxon pared determinou o valor P exato para as diferenças entre a altura da recessão gengival antes e após o tratamento cirúrgico (α = 0,05). Uma média de 97,9% (± 5,6%, p < 0,0001) de cobertura radicular após o tratamento ocorreu, e 73 das 85 recessões apresentaram cobertura radicular completa após 12 meses. O tratamento das recessões gengivais classe I e II de Miller resultou em recobrimento radicular superior a 99 e classe III superior a 95% (p < 0,0001). A série de casos apresentada relata a eficácia de uma técnica cirúrgica modificada promovendo mais de 95% de cobertura radicular após 12 meses em múltiplas recessões gengivais Classe I, II e III de Miller. Ensaios controlados randomizados cegos bem desenhados são necessários para validar a técnica proposta.

## Introduction

One of the main objectives of periodontal plastic surgery is the coverage of exposed roots. Gingival recessions may be related to esthetic problems, dentin hypersensitivity, non-carious cervical lesions, and root caries and may even cause difficulties in teeth hygiene. The success and prognosis of root coverage are associated with gingival recession, local and systemic factors, the surgical technique, and the surgeon’s skill ^1^ ^,^ ^2^ ^,^ ^3^ ^,^ ^4^ ^5^ ^).^

The gingival recession classification allows us to glimpse the possibility of complete root coverage ^6^ ^,^ ^7^. Complete coverage may be predicted in RT1 gingival recession ^6^ or Miller Class I and II ^7^. The interproximal bone loss reduces the possibility of complete coverage in RT2 gingival recessions or Miller class III ^4^ ^,^ ^6^ ^,^ ^7^. The complete root coverage ranges from 42.8% to 90.5% in class III Miller’s recessions ^4^. Multiple gingival recessions also postulate as a challenge to root coverage, once associated with a more significant avascular surface area than unitary recessions and, frequently, to recessions with different heights ^8^. 

The subepithelial connective tissue graft (SCTG) technique provides the highest predictability of root coverage and increases keratinized mucosa height and thickness ^2^ ^,^ ^3^ ^,^ ^4^. After the SCTG description in 1985, some authors proposed several modifications to the original technique, such as the kind of flap and its design, the presence of relaxing incisions, and different suture techniques ^9^ ^,^ ^10^ ^,^ ^11^. 

Nowadays, beyond complete root coverage, techniques that result in excellent aesthetic results without postoperative scar formation are sought. In this context, tunneling techniques are widely performed in periodontal and peri-implant plastic surgery. A published systematic review and meta-analysis recently described complete root coverage in only 57.5% of gingival recessions using the tunnel technique ^12^. Some surgical maneuvers are essential to the success of root coverage, from the incision to the suture technique. Previous authors have recommended suturing the graft in the flap resulting in mobile and unstable anchorage, unable to promote coronal traction of the graft due to the lateral stabilization ^11^ ^,^ ^13^ ^,^ ^14^. We believe that fixed anchors on the adjacent teeth may ensure greater graft stability, favoring angiogenesis and the success of the root coverage.

Aiming to increase the previsibility of the root coverage of the tunneling technique, we designed a new strategy for stabilizing the graft in the bed through an innovative suture technique that favors angiogenesis and repair. Therefore, we report a case series of the modified tunnel technique with connective tissue graft for the root coverage of multiple Miller Class I, II, and III gingival recessions.

### Case series

**Figure 1:**
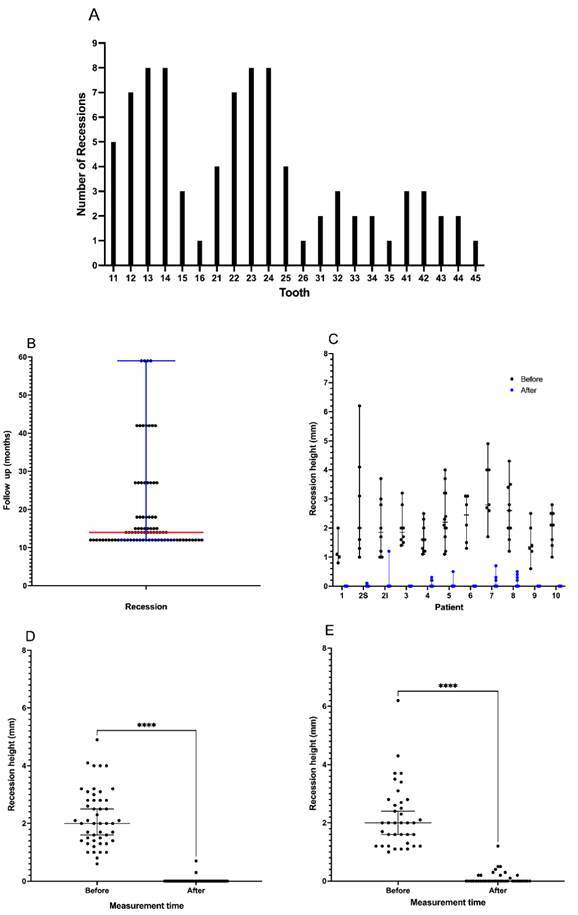
(A) Frequency distribution of recessions per tooth. (B) Recession height before (black) and after (blue) treatment in millimeters. Each point represents one recession with each patient's median, minimum, and maximum values. 2S and 2I represent the recessions for patient 2 on the superior (S) and inferior (I) arch. The height (mm) of all gingival recessions on patients #1, #2S, #3, #6, #9, and #10 was zero after at least 12 months of follow-up. (C) The follow-up period of each gingival recession; the red line is the median (14 months); the blue line is minimum = 12, and maximum = 59 months. Height of Miller class I and II gingival recessions (D) and Miller class III (E) gingival recessions before and after surgical treatment; **** (p < 0.0001, paired Wilcoxon test).

DMG performed all the clinical procedures in this case series conducted at his prived clinic at Belo Horizonte, Minas Gerais, with the patient's consent. Ten patients (2 males and 8 females; age range: 18 to 42), non-smokers, systemically and periodontally healthy, presented to the appointment with dentin hypersensitivity and esthetic complaints due to gingival recession on 85 teeth. Gingival recession was more prevalent on teeth 13, 14, 23, and 24 (Figure 1A); patient #2 presented recessions in both arches. The superior arch presented the highest frequency of interventions (75%) compared to the inferior arch. Teeth 13,14,23, and 24 received 38% of the procedures, 35 out of 85 recessions. The less frequent teeth treated were 16,26, 35, and 45.

The recession height was measured from the cementoenamel junction (CEJ) to the gingival margin (GM), and the width was measured 2mm away from CEJ. Recessions ≥ 3 mm were considered wide but narrow when < 3 mm. Measurement occurred through direct inspection using a digital pachymeter (Starrett Brazil, São Paulo, São Paulo, Brazil; range: 150 mm; resolution: 0.01 mm). The same evaluator carried out all exams and repeated them thrice. The arithmetic average of the three values was considered as the result. Descriptive statistics were applied to calculate the median, minimum and maximum values of the 85 recessions; the data did not pass the Shapiro-Wilk normality test, and pared Wilcoxon test determined the exact P-value for the differences among the median of the gingival recession before and after surgical treatment, considering α = 0.05. The effectiveness of the treatment was expressed as a percentage of the covered recession after the follow-up period. All data were tabulated on an electronic spreadsheet and evaluated on Prism 9.1.0 (GraphPad Software, LLC).

Considering the Miller recession’s classification, 48 were class I and II, and 37 were class III; among the class I and II recessions, 6 out of 48 occurred on the inferior arch; but for class III recessions, 15 out of 37 occurred on the inferior arch suggesting the prevalence of more severe recession on the inferior arch. Narrow recessions (< 3mm), 67 out of the 85, were more frequent than wide ones (≥ 3mm); 51 out 67 of narrow recessions were on superior arch; on the other hand, 13 out 18 of wide recessions were also on the superior arch.

### Surgical technique description

**Figure 2:**
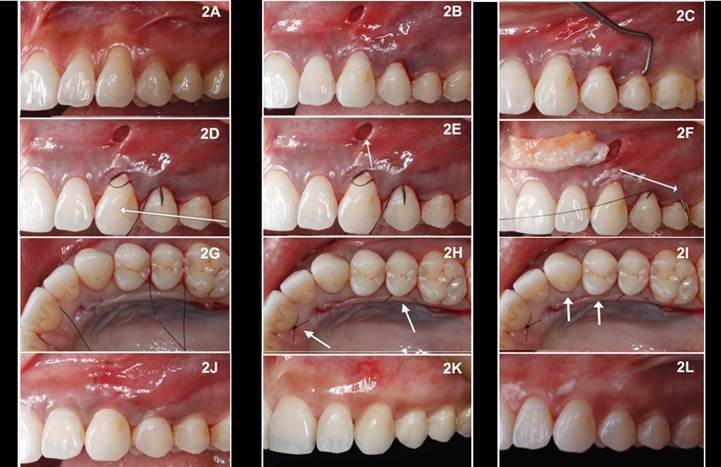
Step-by-step photographs of the modified surgical technique with connective tissue graft for the root coverage of multiple gingival recession. 2A) a representative photograph of the multiple gingival recessions. 2B) vertical maxillary incision of 3 mm in the alveolar mucosa on the mesial portion of the most anterior tooth to be covered. 2C) a full flap was performed towards the papillae and the gingival margin, showing the position of the tunneling instrument performed in the tunnel until the palatine portion. 2D and 2E) the suture (Black Nylon 5-0, Tech Suture, Bauru, São Paulo, Brazil) to stabilize the graft in the receptor bed, began passing the back of the needle (so no tissue was pinched) through the distal portion of the adjacent tooth to the receptor area, from palatal to buccal, without puncturing the papilla; still, with the same orientation, the needle was passed, tooth after tooth, through the buccal gingival margin, until exiting in the vertical access incision, where the graft will be future embedded. 2F) the needle passed through the graft in one of its extremities and then, the way back, until leaving through the mesial and palatal portion of the tooth on which the suture procedure started. 2G) the same suture procedure was performed on the other graft’s extremity using another suture thread, now anchoring it on the mesially located tooth to the grafted area; then, the graft was embedded, pulling the threads located on the teeth adjacent to the grafted area creating two independent suture points. 2H) these sutures were anchored in the adjacent teeth, different from the technique described in the literature, which sutures the graft to the flap. 2I) suspensory sutures (Blue Nylon 6-0, Tech Suture, Brazil) were performed on the teeth to be covered by the graft in a way that the needle punctured only the graft, and the knots were secured at the tooth’s palatal aiming to stabilize the middle portion of the graft; a clinical probe assisted the traction of the graft to coronal positioning easing the punction of the graft. 2J) seeking to cover the graft’s exposed portion, a diagonal sling suture was performed in the flap, granting a coronal pulling of the buccal tissue and positioning the gingival margin beyond the cementoenamel junction (CEJ). 2K and 2J) 14 days and one-year post-surgery photographs.

Figure 2A presents a representative photograph of the multiple gingival recessions. After local anesthesia, a vertical maxillary incision of 3 mm was done in the alveolar mucosa on the mesial portion of the most anterior tooth to be covered (Figures 2B, 3A). Seeking to cover all the necessary areas, other vertical incisions, smaller than the first, could be made in the alveolar mucosa for favorable insertion of the tunneling instruments. After the first incision, using tunneling instruments (Touchgrip®, America Dental Systems, Vaterstetten, Germany), tissue division occurred from the incision, performing a mesiodistal movement until reaching the last molar’s region (Figure 3B). The tunnel is extended at least one tooth beyond the teeth requiring root coverage to mobilize the flap and facilitate coronal repositioning (Figure 3B). Then, a supraperiosteal division of the tissue was performed in all its extension until 3 mm of the gingival margin. Using tunneling instruments, a full flap was performed towards the papillae and the gingival margin (Figures 2C and 3C show the position of the proximal tunneling device used to perform the tunnel). The papillae were displaced using specific tunneling instruments (Maximus Instruments, Contagem, Minas Gerais, Brazil) until the palatine portion (Figure 2C).

**Figure 3:**
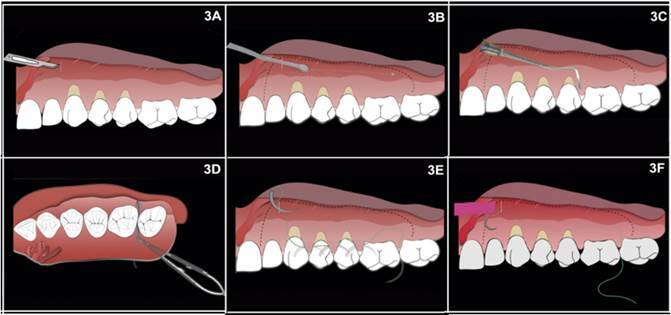
(A-F). Drawings illustrating and detailing the main steps of the modified surgical technique with connective tissue graft for the root coverage of multiple gingival recession. 3A) Drawing representing gingival recessions in teeth 23, 24, and 25. A vertical maxillary incision of 3 mm (white line) is done in the alveolar mucosa on the mesial portion of the most anterior tooth to be covered (tooth 23). 3B) Using tunneling instruments, a supraperiosteal division occurs from the vertical incision, performing a mesiodistal movement until reaching the last molar’s region (tooth 26). The division of the tissue is performed in all its extension until 3mm of the gingival margin. The tunnel is extended at least one tooth beyond the teeth requiring root coverage (teeth 22 and 26) to increase the mobility of the flap and facilitate coronal repositioning (black dashed line). 3C) A full flap is performed towards the papillae and the gingival margin using the designed tunneling instrument. The papilla is displaced using specific tunneling instruments until the palatine portion. 3D) The suture, aiming to stabilize the graft in the receptor bed, begins passing the back of the needle (swage of the needle) through the distal portion of the adjacent tooth to the receptor area (tooth 26), from palatal to buccal, to avoid puncturing the papilla. 3E) Still, with the same orientation, the needle is passed, tooth after tooth, through the buccal gingival margin, until exiting in the vertical access incision, where the graft will be future embedded. The green line corresponds to the forward way of the suture thread and shows that it is under the flap. 3F) After exiting through the vertical incision, the needle punctures the graft on the extremity chosen to be placed distally.

**Figure 3:**
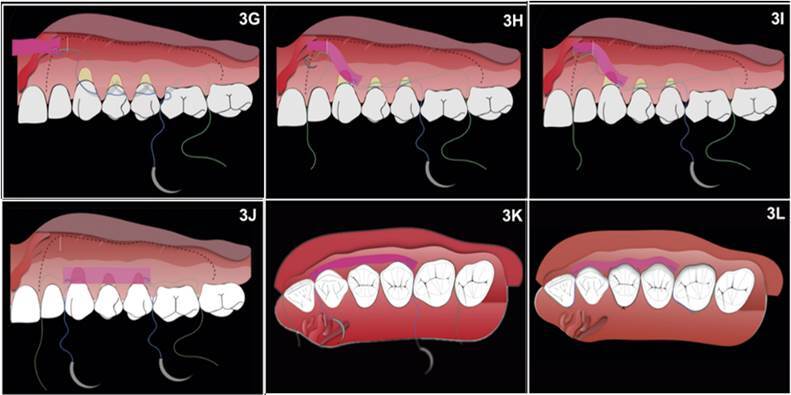
(G-L). After the needle passes through the graft in one of its extremities, it returns until exiting through the mesial/palatal portion of the tooth on which the suture procedure started (tooth 26). The blue line corresponds to the backway of the same suture thread. 3H- As in the distal portion, the suture starts passing the back of the needle (swage of the needle) through the mesial of the adjacent tooth to the receptor area (tooth 22), from palatal to buccal, without puncturing the papilla and coming out in the vertical incision. I - It returns after the needle exits the vertical incision and punctures the graft on the mesial extremity. The brilliant green line corresponds to the forward way of the suture thread, and its opaque portion is under the flap. The brilliant blue line corresponds to the backway of the same suture thread, and its opaque portion is under the flap. J - After the needle punctures the mesial extremity of the graft, it makes its way back until exiting through the distal/palatal portion of tooth 22, on which the suture procedure started. K - In occlusal view, it is possible to visualize the suture extremity on the adjacent teeth' palatal (teeth 22 and 26). Before closing the knot, it is possible to slightly move the graft, stretching the threads and placing it in its best mesiodistal position. L - After stabilizing the knots placed on the adjacent tooth to the receptor area, the most central tooth (tooth 24) receiving the graft will support the sling suture, aiming to pull it coronally and increase its stability. Additional sling sutures may be required.

Curettes and rotary instruments planned the root. The same technique was performed on the mandible, but paying attention when dealing with the mental nerve is crucial. If the access incision coincides with the mental nerve region, it must be moved 10 mm to the mesial, allowing safer access. Furthermore, the tunneling must be done coronally to the foramen. After preparing the receptor bed, the graft was harvested from the palate through the de-epithelialized gingival graft (DGG) technique or linear incision (SCTG), with a thickness of around 1.5mm, measured with a periodontal probe. The suture (Black Nylon 5-0, Tech Suture, Bauru, São Paulo, Brazil), aiming to stabilize the graft in the receptor bed, began passing the back of the needle (so no tissue was pinched) through the distal portion of the adjacent tooth to the receptor area, from palatal to buccal, without puncturing the papilla (Figure 3D). Still, with the same orientation, the needle was passed, tooth after tooth, through the buccal gingival margin, until exiting in the vertical access incision, where the graft will be future embedded (Figures 2D, 2E, 3E). The needle passed through the graft in one of its extremities (Figure 3F) and then, the way back, until leaving through the mesial and palatal portion of the tooth on which the suture procedure started (Figures 2F, 3G). The same suture procedure was performed on the other graft’s extremity using another suture thread, now anchoring it on the mesially located tooth to the grafted area (Figure 3H, 3I). Then, the graft was embedded, pulling the threads on the teeth adjacent to the grafted area creating two independent suture points (Figures 2G, 3J, 3K). These sutures were anchored in the adjacent teeth, different from the technique described in the literature, which sutures the graft to the flap (Figure 2H). Suspensory sutures (Blue Nylon 6-0, Tech Suture, Brazil) were performed on the teeth that received the graft in a way that the needle punctured only the graft, and the knots were secured at the tooth’s palatal (Figure 2I, 3L), aiming to stabilize the middle portion of the graftthe graft. Afterward, seeking to cover the graft’s exposed portion, a diagonal sling suture was performed in the flap, granting a coronal pulling of the buccal tissue, positioning the gingival margin beyond the cementoenamel junction (CEJ) (Figure 2 J, 3L). 

Two platelet-rich fibrin (PRF) membranes were used, and an acrylic plaque of 1mm thickness and clamps positioned on the distal of the last molars on both sides were delivered to the patient without interfering with occlusion to protect and optimize the palatal wound healing. Patients were instructed to avoid brushing the operated regions for around four weeks (Figure 2K). Chlorhexidine mouthwashes (0.12%) were prescribed for 30 days, and the sutures were removed after 14 days. 

## Results

**Figure 4:**
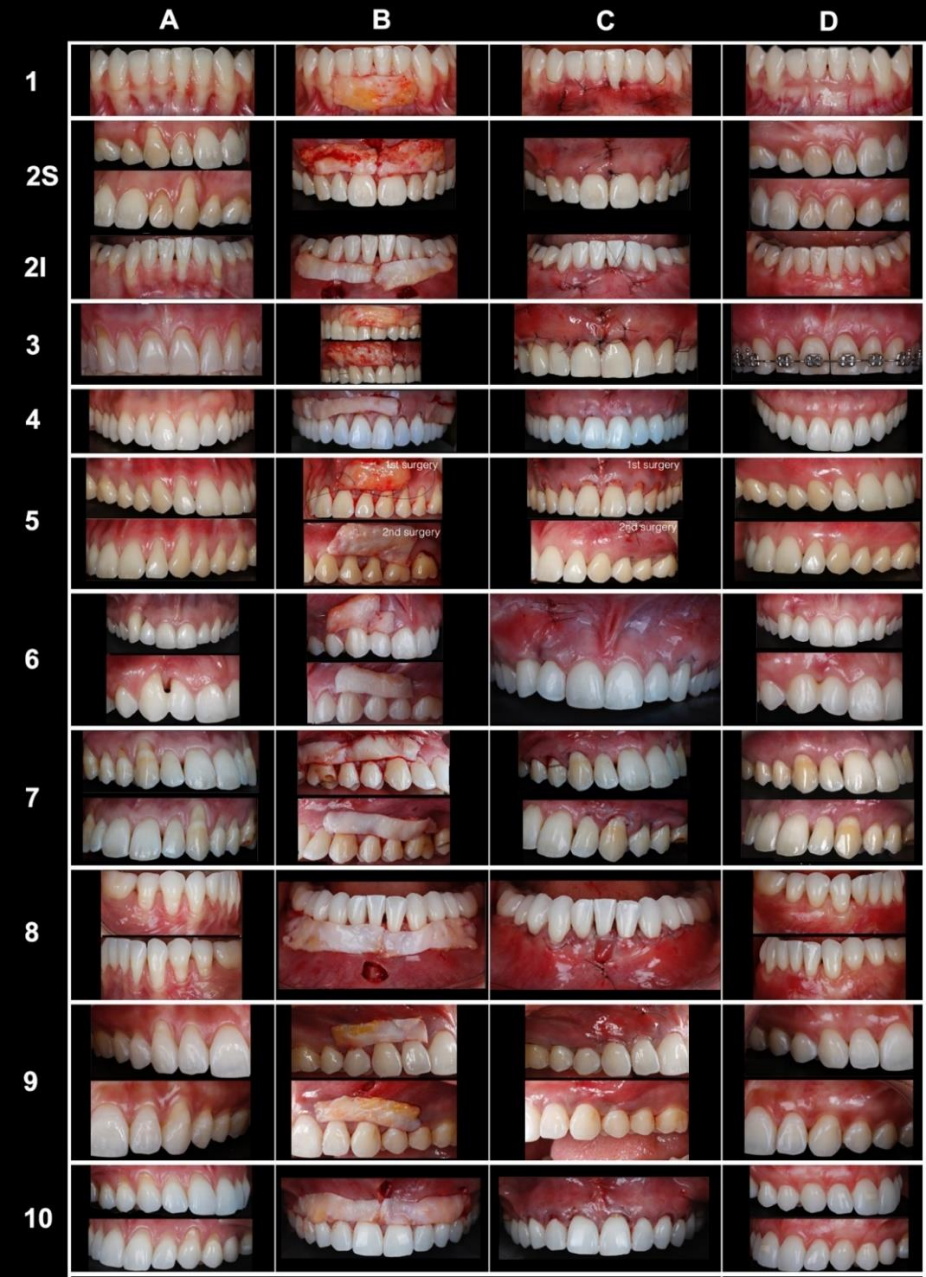
Photographs of the 10 treated cases (lines) showing the pre-operative step (A), the trans-surgical phase showing the position of the connective tissue graft before introduction in the vestibular incision (B), the immediate post-surgery (C), and the follow-up from 12 - 59 months (D).

The patient follow-up varied from 12 to 59 months (Figure 2L), with the median in 14 months. Patients #4, #5, #6, #8, and #9 were followed up for at least 12 months; patient #2I for 14 months; patient #10 for 15 months; patient #7 for 18 months; patient #3 for 27 months; patient #2S for 42 months; and patient #1 for 59 months. Among 85 recessions treated, 37 (44%) were followed up for 12 months, and 64% were followed up for 14 months or more (48 recessions). Patients #2S and #1 were followed up for 42 and 59 months, respectively. Figure 4 presents the 10 treated cases showing the pre-operative step, the trans-operative phase with the gingival graft before tunneling, the immediate post-surgery phase, and the follow-up from 12 - 59 months.

The recession height for each patient’s tooth before and after treatment is shown in figure 1B. Patient #5 presented the highest number of recessions, 12, located on the superior arch, with the recession height ranging from 1.1 to 4.0 mm and a median of 2.2. The smaller height was 0.8 mm for patient #1 on the inferior arch (class I narrow lesion), and the highest was 6.2 mm for patient #2 on the superior arch (class III wide lesion). 

The effectiveness of the treatment is evidenced in Figure 1C. Among 85 recessions, the technique described here provided an average of 97.9% root coverage (SD = 5.6%). Complete root coverage was achieved in 87.1% of sites, two recessions presented 90.0 to 99.0% recovering, and nine presented 80.0 to 89.0%. The difference between mean root coverage in the low (96.05%, CI: 92.31% - 99.79%) and upper (98.52%, CI: 97.44% - 99.59%) arches was not significant (Mann-Whitney test, p=0.210). Patient 2I (tooth 31), with one narrow class III recession, presented a recovery of 68.0%. All recessions of five patients (#1, #3, #6, #9, and #10) stayed 100.0% covered (Figure 4) for at least 12 months. 

For the 48 Miller class I and II gingival recessions, root coverage percentage varied from 82.0% to 100.0%, with an average of 99.6% (SD=2.6%); 47 out of the 48 recessions obtained 100.0% root coverage, representing 97.9% of the Miller class I and II gingival recessions. Only one recession achieved 82.0% of root coverage. Six of 48 Miller class I and II gingival recessions were located on the lower jaw, which achieved complete root coverage. The only recession that obtained 82.0% of root coverage was 4 mm in height but narrow. Wilcoxon test showed a very significant reduction in the recession height after the treatment (p < 0.0001) for the Miller class I and II gingival recessions (Figure 1D).

On the other hand, in the 37 Miller Class III gingival recessions, the root coverage percentage varied from 68.0% to 100.0%, with an average of 96.0% (SD = 7.4%); 27 out of the 37 recessions achieved complete root coverage, representing 72.9% of Miller class III gingival recessions; 9 out of the 37 recessions obtained more than 82.0% root coverage. Of the 37 Miller class III gingival recessions, 15 were located on the lower jaw, resulting in 94,5% root coverage (SD=9.3%). Two of these were wide and measured 4.3 mm and 3.4 mm, with 88.0% and 100.0% root coverage, respectively. Wilcoxon test showed a significant reduction in the recession height after the treatment (p < 0.0001) for the Miller class III gingival recessions. (Figure 1E). 

From 85 gingival recessions, 37 were treated using SCTG. Of these, only two did not achieve 100.0% root coverage, resulting in 94.6% of the sites with complete root coverage; it is essential to underline the 27 gingival recessions (72.9%) treated using SCTG were Miller class I and II gingival recessions, while the other 10 (27.1%) were Miller class III gingival recessions. Forty-eight gingival recessions were treated with DGG and, of these, ten did not achieve 100.0% root coverage, resulting in 79.2% of sites with complete root coverage; from the 48 gingival recessions treated with DGG, 21 (43.0%) were Miller class I and II gingival recessions, and 27 (56.0%) were Miller class

## Discussion

Complete coverage may be predicted in Miller`s Class I and II or Cairo`s RT1 gingival recession (6, 7). However, unitary and multiple RT2 gingival recession or Miller Class III ^6^ ^,^ ^7^ challenge root coverage procedures. Besides, the loss of interproximal insertion may not be a limiting clinical condition for complete root coverage ^3^ ^,^ ^4^ ^,^ ^7^ ^,^ ^15^. Several factors must be considered to obtain complete root coverage in these situations ^3^ ^,^ ^4^ ^,^ ^15^ ^,^ ^16^. Mandibular gingival recession defects also present additional significant challenges, such as shallow vestibule, abnormal frenal attachments, thin gingiva, lack of keratinized gingiva, and thin buccal bone (often dehisced). Multiple gingival recessions turn the challenge even more prominent because of the more significant avascular surface area and recessions with different heights and widths ^8^ ^,^ ^16^ ^,^ ^17^.

In 2011, pre-surgical and surgical variables were evaluated in 121 gingival recessions; 47% of them achieved complete root coverage and, according to the authors, soft tissue interproximal integrity, the use of grafts with a thickness of greater than 2 mm, interproximal bone loss not exceeding 3 mm, and an initial recession width not greater than 3 mm explained the results ^15^. Furthermore, these are critical factors for the success of root coverage procedures: distance from the tip of the papilla to the contact point at baseline, distance from bony crest to cementoenamel junction, gingival thickness, flap tension, width, and height of gingival recessions ^4^ ^,^ ^15^ ^,^ ^16^. 

Many limiting factors were present in this case series, yet the root coverage obtained was higher than previously described in the literature. Tavelli et al. ^12^ published a systematic review and meta-analysis, describing a root coverage average of 87.9% in multiple recessions (SD=16.5%) and complete root coverage in only 57.5% of the gingival recessions using the tunnel technique. There was no significant difference between the mean root coverage in the inferior and superior arches in this case series. The literature has demonstrated that the tooth location is a critical factor for the success of root coverage ^12^. 

We believe that some critical surgical maneuvers were crucial to the success of root coverage in this case series, mainly the innovative suture technique. Despite the high relevance of the suture, some previous studies did not detail address this procedure ^18^ ^,^ ^19^ ^,^ ^20^ ^,^ ^21^. First, using suspending sutures, a fixed anchorage with coronal traction is used to stabilize the graft in the teeth positioned at the mesial and distal ends of the grafted area. Then, suspending sutures are also performed on the teeth that must be covered, stabilizing the graft, and pulling it coronally.

Previous authors have recommended suturing the graft in the flap resulting in mobile and unstable anchorage, unable to promote coronal traction of the graft due to the lateral stabilization ^11^ ^,^ ^13^ ^,^ ^14^. On the other hand, the fixed anchors on the adjacent teeth ensure greater graft stability and intimate contact of the graft with the receptor bed, favoring angiogenesis and promoting earlier and more predictable graft incorporation. Furthermore, the innovative suture technique allows the more coronal positioning of the graft, enabling better tissue filling in the interproximal regions, mainly benefiting the treatment of RT2 recessions.

Other maneuvers in the present case series also contributed to the success of root coverage. The buccal incision on the alveolar mucosa presents advantages such as easy access to perform partial-thickness flap on all apical extensions, which makes it easier to introduce the tunneling instruments needed for the full-thickness flap on the papillae region until 3mm apically to the gingival margin; contributes to the insertion and adaptation of the graft, minimizing the risk of papilla rupture and gingival margin damages. In the vestibular incision subperiosteal tunnel access (VISTA) technique ^18^ ^,^ ^22^, a subperiosteal tunnel has been elevated in all its extensions, extending from the vestibule to the gingival margin, increasing the difficulty for a more coronal positioning of the gingival margins. Disadvantages of the tunneling technique with intrasulcular incisions must include increased difficulty in releasing the flap apically and dislocating it coronally; releasing the papillae until its palatal portion; inserting the graft through the gingival margin, increasing the risk of traumatizing the tissue and the rupture of the papilla, which can lead to undesirable root coverage’s results and scar tissue formation ^18^ ^,^ ^22^.

Previously, the tunnel technique was modified to separate interproximal gingival papilla from the bone tissue, allowing a more coronal positioning of the soft tissues and promoting root coverage ^17^ ^,^ ^19^. This surgical step has been performed via intrasulcular incision, which can hinder the mucoperiosteal release of the interproximal tissues until the palatal portion of the papilla ^8^ ^,^ ^17^ ^,^ ^19^. However, in the present work, for the mucoperiosteal releasing of the interproximal tissue, we used the proximal tunneling device designed by DMG (patent pending, Fig 2C), which was introduced through the vertical buccal incision. It facilitates surgical maneuvers, enabling a more coronal positioning of the CTG in the interproximal region, improving the papilla support mainly in RT2 recessions ^8^.

In summary, the modifications proposed in the present work seem to improve the success of the tunnel technique resulting in a better previsibility of the root coverage. Well-designed, controlled, randomized, and blinded clinical trial is needed to validate the proposed surgical technique.
